# Rapid and accurate electrochemical sensor for food allergen detection in complex foods

**DOI:** 10.1038/s41598-021-00241-6

**Published:** 2021-10-21

**Authors:** Madanodaya Sundhoro, Srikanth R. Agnihotra, Nazir D. Khan, Abigail Barnes, Joseph BelBruno, Lukasz Mendecki

**Affiliations:** Allergy Amulet, 600 Suffolk Street, Suite 268, Lowell, MA 01854A USA

**Keywords:** Analytical chemistry, Electrochemistry, Materials chemistry

## Abstract

Food allergies are estimated to affect about 2–5% of adults and 6–8% of children, globally. Currently, the most effective strategy for food allergy management is stringent avoidance of the offending allergen. Unlike other major food allergens, soy is uniquely challenging to avoid due to its prevalence and insidiousness in a wide variety of foods, such as infant formulas. Recently, we demonstrated a simple, accurate, and consumer-friendly sensor using molecularly imprinted polymers (MIPs) for rapid detection of soy allergenic tracers in complex food matrices at clinically relevant levels. In this work, we build on these findings by subjecting MIP-based soy allergen sensors to test trials in 42 different food products, representing over 300 ingredients. Foods were selected based on their compositional complexity to capture a wide range of preparatory methods and processing conditions. In each case, the Allergy Amulet correctly reported on the presence or absence of soy allergen tracer in investigated samples and were subjected to immunoassay confirmatory analysis. The outcome of this research will help resolve persistent difficulties with commercial technologies in detecting allergenic tracers with minimal cross-interference in foods, and will give those with soy allergies the ability to easily, rapidly, and accurately identify and avoid foods with soy allergens.

## Introduction

Soybean is one of the most common sources of dietary protein due to its reported health benefits, functional properties, and high nutritional value^1,2^. Soy is typically introduced into the diet early in life, often in the form of infant formula, as a substitute to human or cow milk for lactose intolerant infants^3,4^. Despite its reported nutritional benefits, soy is also an important source of food allergens. Soy allergy is among the eight most common forms of food allergy, and in severe cases it can trigger life-threatening anaphylaxis^5^. The estimated prevalence of soy allergy ranges from 0.8 to 1.2% in children and from 0.3 to 0.4% in adults, currently affecting approximately 1.9 million Americans, including 0.4 million children^6,7^. Since soy is nearly omnipresent in processed foods today, consuming soy-based products is essentially unavoidable unless one makes a concerted effort to read labels carefully, and effectively communicates the allergy to those preparing one’s food—even then, the risk of inadvertent ingestion remains.

Currently, no preventative solutions are available for soy allergy sufferers other than strict avoidance. In response, countries have developed legislations and allergen management strategies, requiring manufacturers to identify allergen ingredients on labels to alert consumers on the presence or absence of allergen. In the US, the Food Allergen Labeling and Consumer Protection Act (FALCPA) identifies eight food allergens, including soy, milk, egg, peanut, shellfish, fish, wheat, and tree nuts, that must be identified on the food label^8^. More recently, sesame was added to this list^9^. Even when assuming strict precautionary measures, consumers face a high risk of accidental allergen exposure from adulterated products, undeclared substances, and cross-contamination. A low-cost, accurate, rapid, and consumer-friendly solution for detecting allergens in foods would help account for these risks through greater food transparency and would provide consumers with greater assurances that their foods are safe.

To date, the most widely employed methods for food allergen detection include enzyme‐linked immunosorbent assays (ELISA), lateral flow devices (LFDs), and polymerase chain reaction (PCR). ELISA and LFDs both use monoclonal or polyclonal antibodies to recognize and capture targeted allergens, while PCR relies on detecting DNA fragments of the allergenic species^10–12^. Although many detection kits based on the ELISA, PCR, and LFDs technologies have been successfully commercialized^13^, these methods present several practical limitations undermining the credibility of test results. Among them is the denaturation and/or degradation of proteins and DNA fragments during food processing, which can yield false negative responses^11,14,15^. In addition, antibodies used in the production of immunological bioassays often demonstrate limited thermal stability^16^, are expensive to manufacture, and can cross-react^17^ with other matrix components, which may produce false positive or false negative responses^11^. While LFD strips are arguably the closest assay to a consumer device in terms of simplicity and ease of use, these tests often demonstrate low detection accuracy^18^, requiring multiple samples to verify accuracy. Additionally, changes in food viscosity and texture are known to strongly influence the accuracy of LFD strips in food allergen analysis^19^. Existing commercial detection systems are accordingly ill-equipped for consumer use, and underscore the need for a consumer device capable of rapidly and accurately detecting common allergenic ingredients on-site in food samples.

Recently, we demonstrated the first application of molecularly imprinted polymers (MIPs) to achieve electrochemical detection of soy in complex foods, through detecting a soy allergen marker: genistein^20^. These sensors correctly reported on the presence of soy in food samples subjected to both MIP and LFD measurements. Herein, we carried out the first demonstration of imprinted polymer technology detecting allergens in a large number of foods with varying levels of complexity and homogeneity (e.g., meats, sauces, confectionary, grains, curries, liquids, etc.) prepared under a variety of processing conditions including heat, fermentation, and acidity. For this purpose, we selected 42 food products representing store purchased foods and restaurant products. Together, these foods consist of over 300 ingredients (Supplementary Table S1). In each case, our technology correctly reported on the presence or absence of soy in food samples subjected to the standard LFD allergen detection measurements, demonstrating the effectiveness of our sensors in a diverse range of chemical environments, and the potential of MIP-based technology as a new benchmark for rapid and accurate allergen detection applications.

## Materials and Methods

Genistein was purchased from BOC Sciences (Shirley, NY). Ortho-phenylenediamine (o-PD), catechin hydrate, chrysin, acetic acid, and sodium acetate were purchased from Sigma-Aldrich (Milwaukee, WI). Amygdalin and juglone (5-hydroxy-1,4-napthquinone) were purchased from Alfa Aesar (Tewksbury, MA). Denatured ethanol (5% IPA, 5% *n*-propylacetate) was purchased from Oakwood Chemicals (Estill, SC). PBS 10X (pH = 7.4) was sourced from Boston Bioproducts (Boston, MA). All reagents were of analytical grade and were used without further purification. All aqueous solutions were prepared in ultra-pure water (resistance 18 MΩ cm^−1^) obtained from Satorius arium mini plus Ultrapure Water System (Germany). 1× PBS solutions were prepared by performing a 1:10 dilution of 10× PBS in ultrapure water.

Electrochemical experiments were conducted with a PalmSens4 potentiostat (Palm Instruments BV, Netherlands). Carbon ItalSens IS-C Screen Printed Electrodes (SPE) were purchased from PalmSens (Houten, Netherlands) and were used during all electrochemical measurements. Our allergen sensors, which are part of the Allergy Amulet platform detection system, were prepared according to the method previously developed in our laboratory (Supplementary Experimental S1)^20^.

### Food samples

Store purchased goods included soy curls (Butler), soybeans (Soymerica), tofu (Housefoods), soy sauce (Kim Ve Wong), vegetable oil (Hannaford), Roasted Garlic Parmesan Sauce (Ragu), Captain's Wafers Cream Cheese and Chives (Lance), Thousand Island dressing (Ken’s Steak House), soy protein isolate (Now), granola protein (Nature Valley), soy lecithin (Modernist Pantry), Ritz Crackers with Cheese (Nabisco), Lemon Flavor Crème Oreo (Nabisco), Toast Chee Peanut Butter Crackers (Lance), veggie burger (Morning Star Farms), soy flour (Bob’s Red Mill), defatted soy flour (Scratch), Chicken (Not!) (Dixie Diner’s Club), Zante currant raisins (Sunmaid), tikka masala (Patak’s), sesame seeds (McCormick), rice milk (Rice Dream), red wine vinegar (Market Basket), raisins (Sunmaid), Pure butter shortbread (Walkers), peanut oil (Hain), Moroccan tomato sauce (Mina), mayonnaise (Hellman’s), Major Grey chutney (Patak’s), Original macadamia milk (Milkadamia), Growing Years whole milk (Horizon Organic), green salsa (Mrs. Renfro’s), flax milk (Good Karma), fish sauce (Thai Kitchen), Country French with Orange Blossom Honey dressing (Ken’s Steak House), Coffee Mate creamer (Nestle), cashew milk (So Delicious), Breakfast Blend light roast coffee (Green Mountain), and almond milk (Nature’s Promise) were sourced from local supermarkets. Restaurant dishes, including Ming’s Bings Veggie-Filled Bing patty, duck fried rice and garlic ginger bok choy were sourced from Blue Dragon restaurant (Boston, MA). Confirmatory LFDs measurements were performed using a Soy Rapid Kit L25SOY LFD kit purchased from 3 M.

### Food testing method

**Figure 1:**
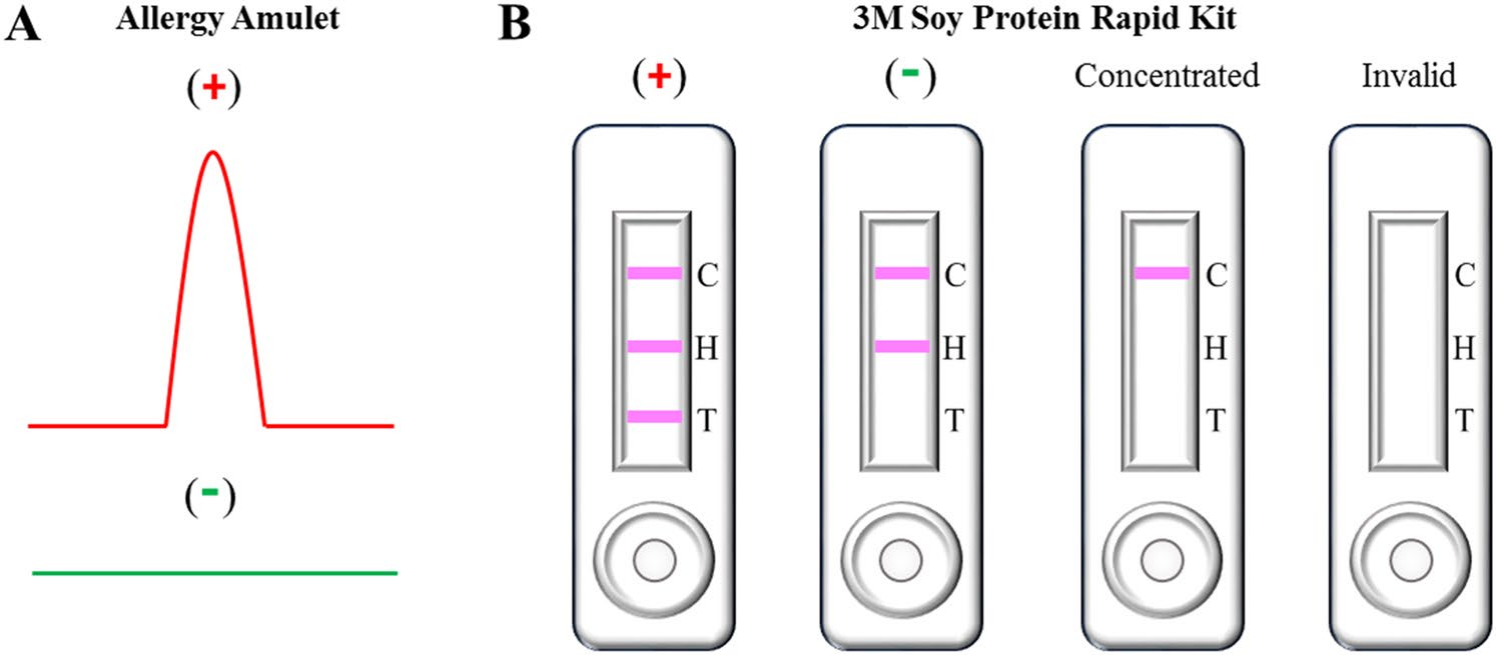
Illustrative responses from: Allergy Amulet kit (**A**) and 3 M Soy Protein Rapid kit (**B**). The Allergy Amulet sensor records a positive reading when a distinct oxidation peak for soy allergen tracer is present at approximately 0.60 V vs Ag/AgCl reference electrode. For the LFD test kits a positive result is visualized by the presence of three lines: a control line (C), a hook line (H) and a test line (T). Negative LFDs results were indicated by the presence of a control and a hook line only. Concentrated LFDs results were indicated by the presence of control line while the invalid results were indicated by the absences of all of three lines.

For each solid food tested, 1 g of food was homogenized using a mortar and pestle (5 min) until a fine powder was obtained. The resulting powder was then mixed with 10 mL of buffer solution and stirred for 15 min. Liquid food samples were prepared by mixing 1 g of food directly with 10 mL of the buffer solution. For both solid and liquid food tests, template-extracted MIP electrodes were inserted into a 10 mL of the buffer solution and equilibrated for 5 min prior to the electrochemical measurements. The electrode was then taken out of the solution and the liquid was removed from the surface. After 1 min incubation with 100 µL of sample solution, the electrodes were subjected to differential pulse voltammetry (DPV) measurements. DPV experimental parameters: scan rate: 50 mV/s; pulse width: 50 ms; and amplitude: 50 mV. All food measurements were run at least in triplicate. Imprinting factor was calculated by dividing signal intensity of MIP with a nonimprinted polymer (NIP) at their peak current maxima (peak position: 0.6 V vs Ag/AgCl reference electrode). A positive response was noted when the sensor reported on the presence of an oxidation peak at approximately 0.60 V vs Ag/AgCl and an imprinting factor above 1.3, which corresponds to oxidative redox transformations of genistein (Fig. 1A)^20^. This electrochemical behavior is consistent with the studies of Popa and Diculescu^21^ and our earlier work^20^.

### LFD testing method

LFDs measurements were carried out according to the 3 M protocol (Fig. 1B)^22^. Briefly, 100 µL of liquid food samples were mixed with 900 µL of 3 M extraction buffer and vortexed for 15 s to aid extraction. 100 µL of the resulting food mixture was then introduced to the 3 M Soy Protein LFD sample well and left to incubate for 11 min. Solid food samples were prepared by homogenizing 1 g of food, using a mortar and pestle, for 5 min. 1.8 mL of the 3 M extraction buffer was then added into a microcentrifuge tube containing 0.2 g of the homogenized samples. The resulting mixture was vortexed for 15 s until the sample was well dispersed prior to 30 s centrifugation at 10,000 rpm using Bio Lion XC-10K. A suspension sample of 100 µL was then introduced into the well of the 3 M Soy Protein LFD and left to incubate for 11 min.

### Cross-reactivity studies

For cross-reactivity measurements, a stock solution with concentration of 1 mg/mL was prepared by dissolving 5 mg of the interferent molecule (amygdalin, juglone, chrysin, or catechin) in 5 mL of ethanol for 15 min. A 10 ppm solution of each interferent was prepared by adding 100 µL of stock solution (1 mg/mL) into a solution containing 900 µL of ethanol and 9 mL of PBS 1×. 10 ppm solutions of both the soy allergen tracer and the analogous molecule were formed by adding 100 µL of soy allergen tracer stock solution and 100 µL of interference stock solution followed by adding 800 µL of ethanol and 9 mL of PBS 1× with 15 min of stirring. DPV measurements were conducted using the same parameters as those used for food testing.

## Results and discussion

Processing and cooking can subject an allergen to denaturation and other conformational changes, which can reduce—but will not necessarily remove completely—its potential to trigger an allergic response. Additionally, allergens may be entrapped or physically constrained to their environment, inhibiting dissolution and/or binding to the selective cavities of the polymer. For the sensor to be effective, it must detect the presence of the allergen regardless of its chemical environment. In this study, we validated the effectiveness of the Allergy Amulet by testing the sensor against 20 different foods known to contain soy and 22 different foods not containing soy. For store-purchased products and restaurant dishes, information on the presence or absence of soy allergen was collected directly from food ingredients used and allergen labels. The integrity of the results requires confirmation of the presence of the allergen in the food by extant allergen detection technology. This was performed using commercially available immunoassay methods (LFDs).

The detection of allergens in food products strongly depends on efficient extraction of soy allergen tracer from complex food matrices. Food processing is known to cause allergen denaturation, conformational changes, aggregation, or chemical modifications^15^. These changes have been reported to strongly influence allergen extractability and antibody recognition of allergenic proteins or DNA fragments in immunoassay or PCR analysis. Conversely, genistein (soy allergen tracer) has been shown to retain its structural stability after being subjected to extensive food processing treatments, including heating and fermentation^23,24^. This approach enables detection of soy in foods even when the DNA and/or the allergenic protein was altered or degraded after food processing. This property is important since it has been reported that soybean can retain its allergenicity even after food processing^25^.

To better understand the impact of food texture and composition on the extractability of soy allergen tracer from foods, we have created a four-point rating scale for grouping different foods by their textural characteristics including crispiness, tenderness, smoothness, toughness, chewiness, creaminess (Table 1). The following ratings were assigned for all tested foods: (1) liquids (soy sauce, vegetable oil, rice milk, red wine vinegar, peanut oil, Original macadamia milk, growing years whole milk, flax milk, fish sauce, Coffee Mate creamer, cashew milk, Breakfast Blend light roast coffee, and almond milk); (2) viscous liquids and emulsions (Roasted Garlic Parmesan Sauce, Thousand Island dressing, tikka masala, Moroccan tomato sauce, mayonnaise, Major Grey chutney, green salsa, Country French with Orange Blossom and Honey dressing); (3) gelatinous substances and soft-solids (tofu, duck fried rice, soy protein isolate, soy lecithin, veggie burger, soy flour, defatted soy flour, Zante currant raisin, raisin, and garlic ginger bok choy); and (4) hard solids (soy curls, soybeans, Captain’s Wafers Cream Cheese and Chives, granola protein, Ritz Cracker with Cheese, Lemon Flavor Crème Oreo, Toast Chee peanut butter crackers, Chicken (Not!), sesame seeds, and Pure butter shortbread) (Table 1). For soy containing foods, we further subcategorized these products based on the origin and source of the soy protein used for their preparation, including soybeans, soy flour, soy protein isolate, soy sauce, tofu, soy lecithin, and soybean oil. We then tested the electrochemical response of MIP-coated electrodes in 10% ethanol, 90% 1× PBS (v/v, pH 7.4) solutions containing 10% by weight of each of the food product representing different textures and soy origins. DPV measurements recorded for soy protein isolate, soy flour, defatted soy flour, tofu, and soybeans showed distinct oxidation peak for soy allergen tracer at approximately 0.6 V (Supplementary Figs. S1–S4) which is consistent with the analyte-centered redox activity of genistein^20,21^. Similarly, positive detection was recorded for soy curls and Chicken (Not!) chunks (Table 1), which contain mainly soybeans or soy flour, respectively. While LFD measurements also reported a positive response for the soybeans, the 3 M LFD test kit failed to inform on the presence of soy allergens in soy protein isolate, soy curls, Chicken (Not!), and tofu (Supplementary Fig. S5A–D), giving rise to a concentrated readout (invalid). The lack of response for LFDs could be caused by protein oversaturation at the detection site, the area at which the biological recognition elements (antibodies, proteins, enzymes, etc.) are immobilized. To overcome this problem, the sample solution—prepared according to the manufacturer’s specifications—had to be repeatedly diluted to achieve the right concentration and enable detection of a soy allergen using LFD. The differences in response characteristics of LFD strips between the soy protein isolate, soy curls, Chicken (Not!) chunks, and the soybeans may be partially attributed to their textural variability. When matured, soybeans can be characterized as a dry and hard solid, which can limit its grinding efficiency using mortar and pestle. Indeed, the other soy-containing hard foods are easier to process using mortar and pestle. As a result, manual processing of soybeans did not yield a well homogenized and homogenous powder, contributing to inefficient extraction of allergenic proteins from soybean particles.

**Table 1:** Detection responses recorded for MIP-coated electrodes and LFDs during food product measurements. A positive test result for both MIP and LFD kit detection confirms that soy allergen is present within a tested sample.

Entry	Food	Brand	Number of ingredients^a^	Presence of soy^a^	Source of soy^a^	Texture^b^	MIP results^c^	LFD results
1	Thousand island dressing	Ken's Steak House	36	Yes	Soybean oil	2	Negative	Negative
2	Almond milk	Nature's Promise	10	No	–	1	Negative	Negative
3	Breakfast blend light roast coffee	Green Mountain	1	No	–	1	Negative	Negative
4	Captain's wafers cream cheese and chives	Lance	30	Yes	Soybean oil	4	Negative	Negative
5	Cashew milk	So Delicious	14	No	–	1	Negative	Negative
6	Chicken (Not!)	Dixie Diner's Club	1	Yes	Soy flour	4	Positive	Concentrated
7	Coffee mate creamer	Nestle	8	No	–	1	Negative	Negative
8	Country French with orange blossom honey dressing	Ken' Steak House	16	No	–	2	Negative	Negative
9	Defatted soy flour	Scratch	1	Yes	Soy flour	3	Positive	Concentrated
10	Duck fried rice	Blue Dragon Restaurant	15	Yes	Soy sauce	3	Negative	Negative
11	Fish sauce	Thai Kitchen	3	No	–	1	Negative	Negative
12	Flax milk	Good Karma	12	No	–	1	Negative	Negative
13	Garlic ginger bok choy	Blue Dragon Restaurant	6	No	–	3	Negative	Negative
14	Granola protein	Nature Valley	11	Yes	Soy protein isolate	4	Positive	Concentrated
15	Green salsa	Mrs. Renfro's	7	No	–	2	Negative	Negative
16	Growing years whole milk	Horizon Organic	7	No	–	1	Negative	Negative
17	Lemon flavor crème oreo	Nabisco	15	Yes	Soy lecithin	4	Negative	Negative
18	Original macadamia milk	Milkadamia	11	No	–	1	Negative	Negative
19	Major grey chutney	Patak's	13	No	–	2	Negative	Negative
20	Mayonnaise	Hellmann's	8	No	–	2	Negative	Negative
21	Veggie filled Ming's Bing	Blue Dragon Restaurant	22	Yes	Soy sauce	22	Positive	Positive
22	Moroccan tomato sauce	Mina	8	No	–	2	Negative	Negative
23	Peanut oil	Hain	2	No	–	1	Negative	Negative
24	Pure butter shortbread	Walkers	8	No	–	4	Negative	Negative
25	Raisin	Sunmaid	1	No	–	3	Negative	Negative
26	Red wine vinegar	Market Basket	1	No	–	1	Negative	Negative
27	Rice milk	Rice Dream	8	No	–	1	Negative	Negative
28	Roasted garlic parmesan sauce	Ragu	25	Yes	Soybean oil	2	Negative	Negative
29	Ritz crackers with cheese	Nabisco	25	Yes	Soy lecithin	4	Negative	Negative
30	Sesame seeds	McCormick	1	No	–	4	Negative	Negative
31	Soy curls	Butler	1	Yes	Soybeans	4	Positive	Concentrated
32	Soy flour	Bob's Red Mill	1	Yes	Soy flour	3	Positive	Concentrated
33	Soy lecithin	Modernist Pantry	1	Yes	Soy lecithin	3	Negative	Concentrated
34	Soy protein isolate	Now	1	Yes	Soy protein isolate	3	Positive	Concentrated
35	Soy sauce	Kim Ve Wong	5	Yes	Soybeans	1	Positive	Negative
36	Soybeans	Soymerica	1	Yes	Soybeans	4	Positive	Positive
37	Tikka masala	Patak's	20	No	–	2	Negative	Negative
38	Toast chee peanut butter crackers	Lance	25	Yes	Soy lecithin, soybean oil	4	Negative	Weak Positive
39	Tofu	Housefoods	4	Yes	Soybeans	3	Positive	Positive
40	Vegetable oil	Hannaford	1	Yes	Soybean oil	1	Negative	Negative
41	Veggie burger	Morning star farms	27	Yes	Soy flour, soy sauce powder	3	Positive	Positive
42	Zante currant raisin	Sunmaid	1	No	–	3	Negative	Negative

^a^As reported in ingredients lists.

^b^Based on scale 1–4 with 1 liquid, 2 viscous liquid and emulsions, 3 gelatinous and soft solid, 4 hard solid.

^c^Based on triplicated electrochemical readings.

Further, LFD measurements did not register a positive response for soy flour samples (Supplementary Fig. S5F). This inconclusive result may be attributed to the increased viscosity of the tested solution, inhibiting the fluid flow across the LFD substrate even after hours of incubation. While diluting the soy flour solution four times enabled the movement of the liquid to the detection area, the device still reported a concentrated result (Supplementary Fig. S5G). The susceptibility of tested LFDs to produce positive responses in soy-rich foods poses practical limitations if such products were to be used in consumer allergen testing applications, as the user would in theory be required to undertake extensive sample processing steps, and run multiple measurements to realize accurate detection.

We then tested the electrochemical response of MIP-coated electrodes for two soy-labelled liquid products: soy sauce and soybean oil. The DPV measurements of soy sauce and soybean oil did not reveal a characteristic response for soy tracer at 0.60 V, but instead generated a smaller signal at approximately 0.80 V for soy sauce containing samples. The observed peak current at higher anodic potentials may be due to the presence of polyphenols in soy sauce^26^. Confirmatory LFDs tests also reported a negative responses for soy sauce (Supplementary Fig. S5H) and soybean oil samples (Supplementary Fig. S5I). These experimental observations can most likely be explained by very low content of allergenic soy protein, resulting from the industrial fermentation processes (e.g., microbial proteolytic enzymes) used for the manufacturing of many soy sauces. Indeed, food processing is known to reduce the allergenicity of soy and wheat proteins in processed foods^27^. Similarly, the industrial processes of refining soybean oil typically involve multiple extraction steps using hot solvents, bleaching, and deodorization, which serve to effectively eliminate the allergenic soy protein from the soybean oil-based products. Additionally, it has been showed that soybean oil is generally safe to consume for soy allergenic individuals^28^. Unlike the positive results, obtaining negative results is quite straightforward in LFDs. In most cases, clear hook and control lines are easily observable. In most foods containing soy allergen, LFD results tend to give concentrated or faint signals on both the hook line and the test line. A positive or invalid result in these cases would be subject to the perception of the interpreter.

Lastly, we studied the response of our sensors when testing soy lecithin. Soy lecithin is a common soy-based additive used in the food industry as an emulsifier, lubricant, antioxidant, and flavor protector. Because soy lecithin is produced from highly refined soy oils, it typically contains insufficient amounts of allergenic soy protein to provoke allergic reactions in most soy-allergic individuals. For example, the Food & Drug Administration (FDA) has granted exemptions regarding the labelling of soy lecithin as an allergen on food products. These exemptions apply when soy lecithin is used directly as a release agent or a component of a release agent applied to food contact surfaces^29^. DPV measurements with MIP-coated electrodes on soy lecithin reported a negative response as evidenced by the lack of an anodic peak at 0.6 V vs Ag/AgCl reference electrode. Like soy flour, LFD tests produced an inconclusive readout—the liquid could not readily travel from the loading well to the detection site (Supplementary Fig. S5J). Although, LFDs have been widely regarded as rapid and portable food tests, these are highly susceptible to the presence of matrix components and overall sample consistency which together can cause pore obstruction, and thus limit the liquid flow. After carrying out additional sample dilutions (tenfold), the resulting “slurry” was of the right consistency to produce a visible readout using immunoassay analysis. The confirmatory LFD strips also reported a negative response, indicating the absence of soy allergenic protein at clinically relevant levels in tested soy lecithin samples (Supplementary Fig. S5K).

### Grocery store foods

After studying the influence of food texture and composition on detection performance of the Allergy Amulet sensor, we focused our attention on expanding the range and number of use cases in food allergen analysis applications. We accordingly selected 21 soy-free and 18 soy-containing store-bought products, each prepared using a different manufacturing process and having a unique composition (Supplementary Table S1). Electrochemical measurements of Captain's Wafers Cream Cheese & Chives sandwich crackers, Toast Chee Peanut Butter Crackers, and Ritz Crackers with Cheese reported a negative response as evidenced by the lack of a characteristic signal for soy allergen tracer at 0.6 V vs Ag/AgCl. As the primary source of soy in these foods is soy lecithin, these results were consistent with our earlier data obtained from soy lecithin measurements. While the LFD tests on the Captain's Wafers Cream Cheese & Chives and Ritz Crackers with Cheese confirmed the absence of soy allergen in each food, the immunoassay reported a slightly positive readout for Toast Chee Peanut Butter Crackers samples (Supplementary Fig. S1L). This result was unexpected, as the only soy-based ingredient in Toast Chee Peanut Butter Crackers identified by the manufacturer and listed on a food label was soy lecithin. The faint positive signal recorded in LFD analysis of Toast Chee Peanut Butter Crackers may be due to the “fatty or smeary” nature and texture of the food. These compositional and textural characteristics have been already reported to influence the accuracy of LFDs in detecting allergens, giving rise to false positive readouts^30^.

Lemon Flavor Crème Oreo is another food containing soy lecithin as its soy source. DPV measurements of Lemon Flavor Crème Oreo sandwich cookies did not report on the presence of soy allergen tracer at 0.6 V, but instead a weak anodic signal was observed at 0.5 V vs Ag/AgCl. The recorded electrochemical signal at a more cathodic potential may be due to the presence of annatto extract in tested cookies. Annatto is a common food dye known to contain redox-active polyphenols^31^. The LFD measurements also did not produce a positive response during testing of Lemon Flavor Crème Oreo sandwich cookies samples, indicating the high accuracy of our MIP-based sensors. Subsequently, we subjected MIP-coated electrodes to food measurements with two soy-containing solid food products: soy burger patties and granola protein bars. Soy flour and soy protein isolate were identified as the primary sources of soy in each respective formulation. Using Allergy Amulet and LFD immunoassays, we correctly identified the presence of soy in both tested samples. These findings were consistent with our earlier test results on soy flour and soy protein isolate. We then tested highly complex semi-liquid food samples (texture scale: 2) including Roasted Garlic Parmesan Sauce and Thousand Island dressing, consisting of 25 and 36 individual ingredients, respectively. The food labels indicated that both of these foods contained soy in the form of a soybean oil. Electrochemical and LFD measurements of these foods demonstrated a negative readout, matching those results obtained from earlier analysis on soybean oil samples.

**Figure 2:**
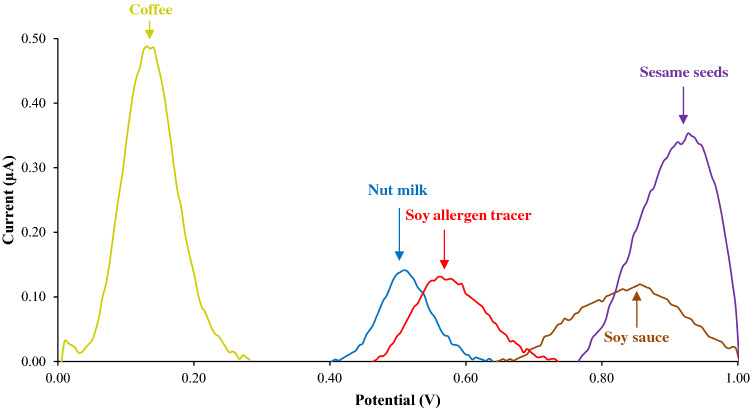
DPV responses for soy allergen tracer (red line), soy sauce (brown line), sesame seeds (purple line), coffee (gold line), nut milk (blue line) 10% by weight in buffer solution containing 10% ethanol in 90% PBS 1×.

Polyphenols, like genistein, are well-known antioxidant molecules found in several foods, herbs, and spices. These species are known to define some of the organoleptic characteristics of foods—e.g., color, flavor, and bitterness. The presence of phenolic moieties in polyphenols, being the main constituents of these compounds, confers their inherent redox activity and their antioxidant characteristics. As these molecules can demonstrate redox activity during electrochemical analysis, we investigated the detection capability of the MIP-based electrodes on molecules that are structurally similar to the soy allergen tracer that are present in significant quantities in foods (Supplementary Fig. S6). We then continued this investigation on a range of soy-free foods known to be rich in polyphenols, including coffee, nut milks (macadamia milk, flax milk and cashew milk), fish sauce, and sesame seeds^26,32–39^. Indeed, electrochemical measurements of these foods reported on the presence of anodic peaks at 0.1 V, 0.5 V, 0.8 V and 0.9 V vs Ag/AgCl reference electrode, respectively (Fig. 2). No characteristic signal associated with the presence of soy allergen tracer (0.6 V) was detected during testing of soy-free foods with high polyphenol content. LFD analysis also reported on the negative response, indicating that such polyphenols had minimal interference on the accuracy of MIP-based sensors. These findings demonstrate that the differences in redox potentials of polyphenolic molecules make it possible to distinguish these analytes electrochemically from soy allergen tracer (Fig. 2). While other soy-free store purchased products including almond milk, rice milk, red wine vinegar, peanut oil, whole milk, Coffee Mate creamer, Pure butter short bread, raisins, and Zante currants may contain small levels of polyphenolic compounds, these foods did not produce any voltametric response in the investigated potential range (0–1 V). This effect might be caused by insufficient levels of these compounds to produce anodic signals in the extracted sample, as well as their redox processes taking place at more negative or positive potentials which fall outside the measurement range. These results demonstrate that our imprinted polymer-based sensors can successfully differentiate soy allergen tracer from other redox active species typically found in foods.

### Restaurant dishes

Restaurant dishes are typically more complex than homemade foods or store-bought products in terms of their number of ingredients and textures. They are also an important category for validation due to the risk restaurant dining poses to allergy sufferers. Therefore, we selected one restaurant dish that was prepared without soy (garlic ginger bok choy) and two dishes prepared with soy (duck fried rice and veggie filled Ming’s Bings Veggie-Filled Bing patty). Each dish was made up of at least seven individual ingredients and represented different cooking and processing methods (see Supplementary Table S1 for breakdown of ingredients). During garlic ginger bok choy measurements, the Allergy Amulet correctly reported on the absence of soy allergen tracer in tested foods subjected to immunoassay measurements (Table 1). Although, both duck fried rice and Ming’s Bing patty have listed soy sauce as one of their ingredients, the Allergy Amulet and LFDs only reported a positive response for the Ming’s Bing patty (Table 1). These results may be explained by the differences in the type^25^ and amount of soy sauce used for preparing each individual dish.

After the original soy-free food samples were tested, they were “spiked” with a 10 ppm solution of the allergenic tracer. The tests were then repeated to confirm the efficacy of the device in the food matrix now containing the allergen. In each case, our detection platform correctly identified the presence of soy allergen tracer in soy-spiked foods, showing minimal background interferences (data not shown).

## Conclusion

In this work, we confirmed the feasibility of MIP-based sensors for soy allergen detection in complex foods. We selected food products that represented a wide range of sources (e.g., store-bought and restaurant dishes) and chose foods that ensured we could distinguish between those dishes containing soy and those that did not. For every food that was known to contain soy, Allergy Amulet correctly detected its presence at clinically relevant levels. To confirm that our sensor was performing at least as well as existing commercial allergen detectors, we then tested those same foods against a lateral flow device (LFD)—one of the key methods for testing allergenic ingredients in commerce. An exact binomial test was used to compare the binary accuracies of the MIP sensor and the LFD kit in detecting the presence of soy allergen, which confirmed the higher degree of accuracy of the MIP (P = 0.007). In particular, our sensors appeared to be superior in testing of highly concentrated soy-based products and foods with higher fat content. Therefore, we have determined that our MIP-based sensors are not only a suitable alternative to other analytical methods frequently used for food allergen testing, but also offers advantages in personal food allergen detection applications and food safety control that allow for detection in a broader range of conditions than were previously deemed possible.

## Supplementary Information

Supplementary Information.
