# Supplementary material for: Rapid and accurate electrochemical sensor for food allergen detection in complex foods
Source: Sci Rep. 2021 Oct 21;11:20831. doi: 10.1038/s41598-021-00241-6 (PMC8531013; doi:10.1038/s41598-021-00241-6)
Supplement: Supplementary file 1 — Supplementary Information. [file 41598_2021_241_MOESM1_ESM.docx]

**Supporting Information**

Rapid and Accurate Electrochemical Sensor foy Food Allergen Detection in Complex Foods.

Madanodaya Sundhoro, Srikanth R. Agnihotra, Nazir D. Khan, Abigail Barnes, Joseph BelBruno, Lukasz Mendecki*

Allergy Amulet, 600 Suffolk Street, Suite 268, Lowell, Massachusetts 01854

**Table of Contents**

[**Experimental S1**. Imprinted polymer synthesis. 2](#_Toc82358461)

[**Table S1**. Foods and their ingredients list. 2](#_Toc82358462)

[**Figure S1**. DPV data of soy protein isolate in 10 mL of 10% ethanol and 90% PBS 1X. 6](#_Toc82358463)

[**Figure S2**. DPV data of soy flour (A) and defatted soy flour (B) in 10 mL of 10% ethanol and 90% PBS 1X. 7](#_Toc82358464)

[**Figure S3**. DPV data of tofu in 10 mL of 10% ethanol and 90% PBS 1X. 8](#_Toc82358465)

[**Figure S4**. DPV data of soybeans in 10 mL of 10% ethanol and 90% PBS 1X. 8](#_Toc82358466)

[**Figure S5**. LFDs data from soy protein isolate (**A**), Chicken (Not!) (**B**), soy curl (**C**), tofu (**D**), soybean (**E**), soy flour (**F**), 4X dilution soy flour (**G**), soy sauce (**H**), soybean oil (**I**), soy lecithin (**J**), 10X dilution soy lecithin (**K**), and Toast Chee peanut butter crackers (**L**). 9](#_Toc82358467)

[**Figure S6.** DPV responses recorded for soy allergen tracer (red line), juglone (blue line), chrysin (purple line), catechin (green line), and amygdalin (gold line) at 10 ppm concentration in 10% ethanol and 90% PBS 1X. 10](#_Toc82358468)

[**Figure S7.** DPV peak currents for the MIP and NIP in 10 ppm of the soy allergen tracer in 10% ethanol and 90% 1X PBS. 11](#_Toc82358469)

# **Experimental S1**. Imprinted polymer synthesis.

Briefly, 1.82 mg of genistein and 40.6 mg of o-PD were dissolved in 50 mL of a 10% ethanol, 90% 0.1 M acetic acid/sodium acetate buffer solution (v/v, pH 5.2) for one hour. The solution was degassed for 15 min, and the SPEs were then placed in a degassed solution for 5 min prior to the polymerization. Electrodeposition of MIP-coatings was carried out for one minute at 1.2 V under nitrogen atmosphere. The soy allergen tracer was extracted from the polymer film by washing the resulting sensors in a solution of 0.1 M NaOH for one hour and left to dry at room temperature for 24 h.

# **Table S1**. Foods and their ingredients list.

| Entry | Food | Brand | Ingredients |
| --- | --- | --- | --- |
|  |  |  |  |
| 1 | Thousand Island Dressing | Ken's Steak House | Soybean Oil, Chili Sauce, Sugar, Vinegar, Salt, Natural Flavor, Spice, Onion Powder, Garlic Powder, Vinegar, Water, Sugar, Sweet Pickle Relish (Cucumber, Sugar, Vinegar, Salt, Mustard Seed, Celery Seed, Onion, Xanthan Gum, Natural Flavor, Red Bell Pepper, Turmeric, Egg Yolk, Contains Less Than 2% Of Salt, Mustard Flour, Propylene Glycol Alginate, Sodium Benzoate, Xanthan Gum, Onion Powder, Onion, Garlic, Garlic Powder, Calcium Disodium EDTA, Red Bell Pepper, Oleoresin Paprika |
| 2 | Almond Milk | Nature's Promise | Almond Milk, Calcium Carbonate, Tapioca Maltodextrin, Sea Salt, Potassium Citrate, Carrageenan, Sunflower Lecithin, Natural Flavor, Vitamin A Palmitate, Vitamin D2, D-alpha-tocopherol, |
| 3 | Breakfast Blend Light Roast Coffee | Green Mountain | Arabica Coffee |
| 4 | Captain's Wafers Cream Cheese and Chives | Lance | Wheat Flour, Niacin, Reduced Iron, Thiamine Mononitrate, Riboflavin, Folic Acid, Palm Oil, Soybean Oil, Canola Oil, Deproteinized Dairy Whey, Sugar, Buttermilk, Corn Syrup Solids, Sodium Bicarbonate, Ammonium Bicarbonate, Monocalcium Phosphate, Salt, Corn Syrup, Cream Cheese, Cheese Cultures, Salt, Xanthan, Carob Bean, Guar Gum, Sodium Caseinate, Spices, Nonfat Dry Milk, Natural and Artificial Flavor, Soy Lecithin, Peanut Oil |
| 5 | Cashew Milk | So Delicious | Filtered Water, Cashews, Canola Oil, Calcium Phosphate, Magnesium Phosphate, Calcium Carbonate, L-Selenomethionine [Selenium], Vitamin A Acetate, Vitamin D2, Zinc Oxide, Vitamin B12, Guar Gum, Kosher Sea Salt, Locust Bean Gum, Gellan Gum, Natural Flavor |
| 6 | Chicken (Not!) | Dixie Diner's Club | Soy Flour |
| 7 | Coffee Mate Creamer | Nestle | Water, Coconut Oil, Sugar, Sodium Caseinate, Monoglycerides, Diglycerides, Dipostassium Phosphate, Natural Flavor, Beta-Carotene Color |
| 8 | Country French with Orange Blossom Honey Dressing | Ken' Steak House | Soybean Oil, Sugar, Water, Distilled Vinegar, Apple Cider Vinegar, Honey, Tomato Paste, Salt, Paprika, Mustard Flour, Xantham Gum, Onion, Garlic, Beetroot Juice Powder, Natural Flavor, Calcium Disodium EDTA |
| 9 | Defatted Soy Flour | Scratch | Defatted Soy Flour |
| 10 | Duck Fried Rice | Blue Dragon Restaurant | White Rice, Brown Rice, Onion, Ginger, Garlic, Duck Egg, Soy Sauce, Chive, Mango, Tamari, Lemon, Cilantro, Fresno, Chili, Red Onion |
| 11 | Fish Sauce | Thai Kitchen | Anchovy Extract (Fish), Salt, Sugar |
| 12 | Flax Milk | Good Karma | Flax milk, Tapioca Starch, Tricalcium Phosphate, Sunflower Lecithin, Natural Flavors, Sea Salt, Vanilla Extract, Gellan Gum, Xantham Gum, Vitamin A Palmitate, Vitamin D2, Vitamin B12, |
| 13 | Garlic Ginger Bok Choy | Blue Dragon Restaurant | Canola Oil, Garlic, Ginger, Chicken Stock, Butter, Sweet Soy Sauce |
| 14 | Granola Protein | Nature Valley | Sugar, Soy Protein Isolate, Canola Oil, Honey, Molasses, Rice Starch, Soy Lecithin, Baking soda, Salt, Natural Flavor, Vitamin E |
| 15 | Green Salsa | Mrs. Renfro's | Jalapeno Peppers, Water, Distilled Vinegar, Corn Starch, Salt, Dried Onion, Spices, Dried Garlic |
| 16 | Growing Years Whole Milk | Horizon Organic | Grade A Organic Milk, Chicory Root Extract, DHA Algal Oil, Choline Chloride, Ascorbic Acid, Tocopherols, Vitamin D3 |
| 17 | Lemon Flavor Creme Oreo | Nabisco | Wheat Flour, Niacin, Reduced Iron, Vitamin B1, Riboflavin, Folic Acid, Palm Oil, High Fructose Corn Syrup, Salt, Baking Soda, Soy Lecithin, Natural Flavor, Citric Acid, Artificial Flavor, Annatto Extract, Canola Oil |
| 18 | Original Macadamia Milk | Milkadamia | Macadamia Milk, Calcium Phosphate, Pea Protein, Natural Flavors, Sunflower Lecithin, Locust Bean Gum, Sea Salt, Gellan Gum, Vitamin A Palmitate, Vitamin D2, Vitamin B12 |
| 19 | Major Grey Chutney | Patak's | Sugar, Mango Chunks, Salt, Ginger, Water, Acetic Acid, Spices, Paprika, Garlic, Natural Ginger Flavor, Peanut, Almond, Cashew, Coconut |
| 20 | Mayonnaise | Hellmann's | Water, Whole Eggs and Egg Yolks, Distilled Vinegar, Salt, Sugar, Lemon Juice Concentrate, Calcium Disodium EDTA, Natural Flavors, |
| 21 | Veggie Filled Ming's Bing | Blue Dragon Restaurant | Red Onions, Sliced Shitake Mushrooms, Watercress, Edamame, Onions, Canola oil, Organic Vinegar, Salt, Citric Acid, Roasted Spicy Pumpkin Seeds, Organic Ginger Puree, Oil (80% Canola, 20% Extra Virgin), Organic Cane Sugar, Non-GMO Tamari Powder, Soy Sauce, Spices, Water, Brown Rice Flour, Tapioca Starch, Olive Oil, Xanthan Gum, Sea Salt |
| 22 | Moroccan Tomato Sauce | Mina | Roasted Peppers, Onions, Extra Virgin Olive Oil, Cilantro, Garlic, Parsley, Spices, Salt |
| 23 | Peanut Oil | Hain | Peanut Oil, Vitamin E |
| 24 | Pure Butter Shortbread | Walkers | Wheat Flour, Butter, Sugar, Salt |
| 25 | Raisin | Sunmaid | Raisin |
| 26 | Red Wine Vinegar | Market Basket | Red Wine Vinegar, Water, Sulfiting Agent |
| 27 | Rice Milk | Rice Dream | Filtered Water, Organic Brown Rice, Safflower Oil, Tricalcium Phosphate, Sea Salt, Vitamin A Palmitate, Vitamin D2, Vitamin B12, Sunflower Oil, Canola Oil |
| 28 | Roasted Garlic Parmesan Sauce | Ragu | Water, Soybean Oil, Cream, Parmesan Cheese, Cheese Cultures, Enzymes, Modified Corn Starch, Enzyme Modified Egg Yolk, Romano Cheese, Salt, Chey, Roasted Garlic, Disodium Phosphate, Xanthan Gum, Yeast Extract, Whey Protein Concentrate, Garlic Powder, Spices, Natural Flavors |
| 29 | Ritz Crackers with Cheese | Nabisco | Unbleached Enriched Wheat Flour, Niacin, Reduced Iron, Thiamine Mononitrate, Riboflavin, Folic Acid, Palm Oil, Canola Oil, Sugar, Whey, Sunflower Oil, Baking Soda, Cheddar Cheese Powder, Pasteurized Milk, Cheese Culture, Salt, Enzymes, Salt, Disodium Phosphate, Soy Lecithin, Natural Flavor, Turmeric Extract, Dried Yeast, Buttermilk, Lactic Acid, Vinegar, Calcium Phosphate, Annatto Extract |
| 30 | Sesame Seeds | McCormick | Sesame Seeds |
| 31 | Soy Curls | Butler | Soybeans |
| 32 | Soy Flour | Bob's Red Mill | Whole Ground Soybeans |
| 33 | Soy Lecithin | Modernist Pantry | Soy Lecithin |
| 34 | Soy Protein Isolate | Now | Soy Protein Isolate |
| 35 | Soy Sauce | Kim Ve Wong | Water, Soybeans, Wheat, Salt, Sugar |
| 36 | Soybeans | Soymerica | Soybeans |
| 37 | Tikka Masala | Patak's | Tomato, Onion, Cream, Yogurt, Whole Milk, Skimmed Milk Solids, Yogurt Culture, Tomato Paste, Canola Oil, Spices, Paprika, Turmeric, Food Starch-Modified, Sugar, Garlic, Ginger, Salt, Lactic Acid, Cumin Seed, Paprika Extract, Dried Cilantro |
| 38 | Toast Chee Peanut Butter Crackers | Lance | Enriched Wheat Flour, Niacin, Reduced Iron, Thiamine Mononitrate, Riboflavin, Folic Acid, Peanut Butter, Soybean Oil, Palm Oil, Canola Oil, Dextrose, Sugar, Salt, Sodium Bicarbonate, Monocalcium Phosphate, Ammonium Bicarbonate, Corn Syrup, Deproteinized Dairy Whey, Cheddar Cheese, Cheese Cultures, Salt, Enzymes, Yellow 6, Soy Lecithin, Pasteurized Milk |
| 39 | Tofu | Housefoods | Water, Soybeans, Calcium Sulfate, Calcium Chloride |
| 40 | Vegetable Oil | Hannaford | Soybean Oil |
| 41 | Veggie Burger | Morning Star Farms | Wheat Gluten, Soy Flour, Vegetable Oil, Egg Whites, Water, Calcium caseinate, Tapioca Starch, Onion Powder, Soy Sauce Powder, Methylcellulose, Cooked Onion and Carrot Juice Concentrate, Salt, Natural Flavor, Soy Protein Isolate, Garlic Powder, Spices, Sugar, Dextrose, Gum Acacia, Whey, Yeast Extract, Xanthan Gum, Potato Starch, Tomato Paste, Onion Juice Concentrate |
| 42 | Zante Currant Raisin | Sunmaid | Zante Currants |

# **Figure S1**. DPV data of soy protein isolate in 10 mL of 10% ethanol and 90% PBS 1X.

**A**

**Figure S2**. DPV data of soy flour (A) and defatted soy flour (B) in 10 mL of 10% ethanol and 90% PBS 1X.

**B**

**Figure S3**. DPV data of tofu in 10 mL of 10% ethanol and 90% PBS 1X.

# **Figure S4**. DPV data of soybeans in 10 mL of 10% ethanol and 90% PBS 1X.


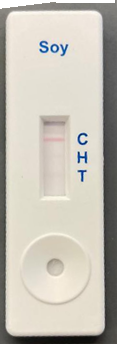

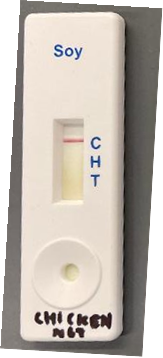

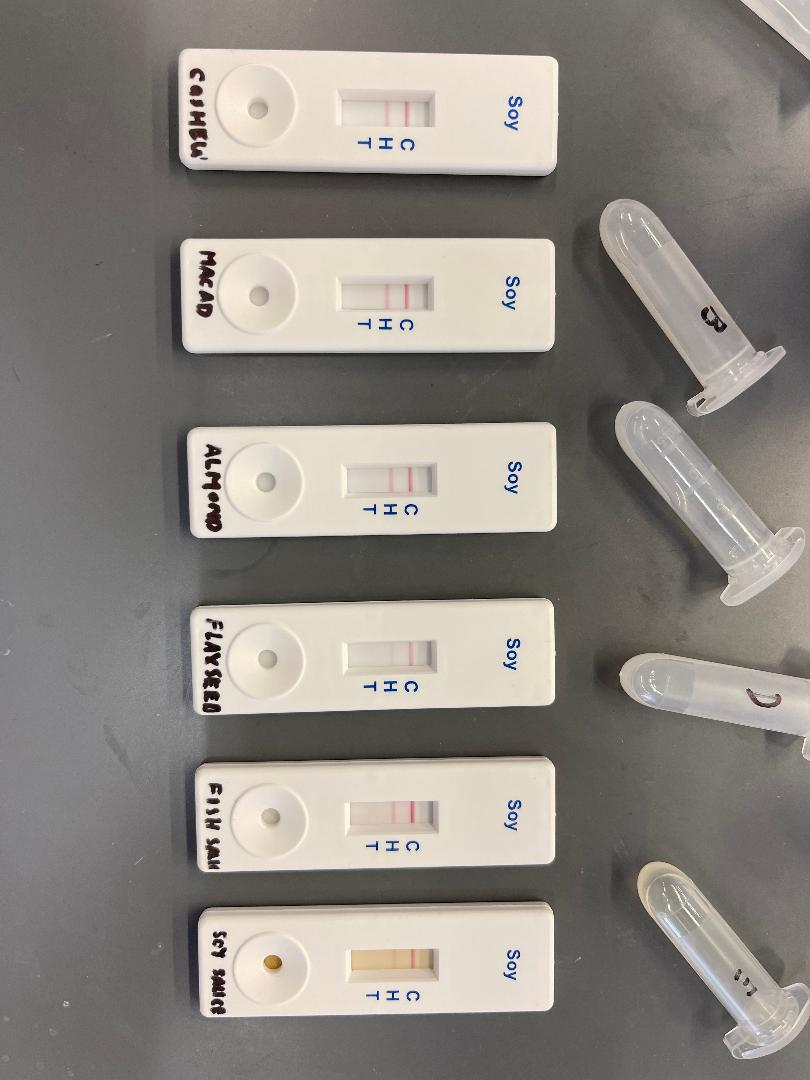

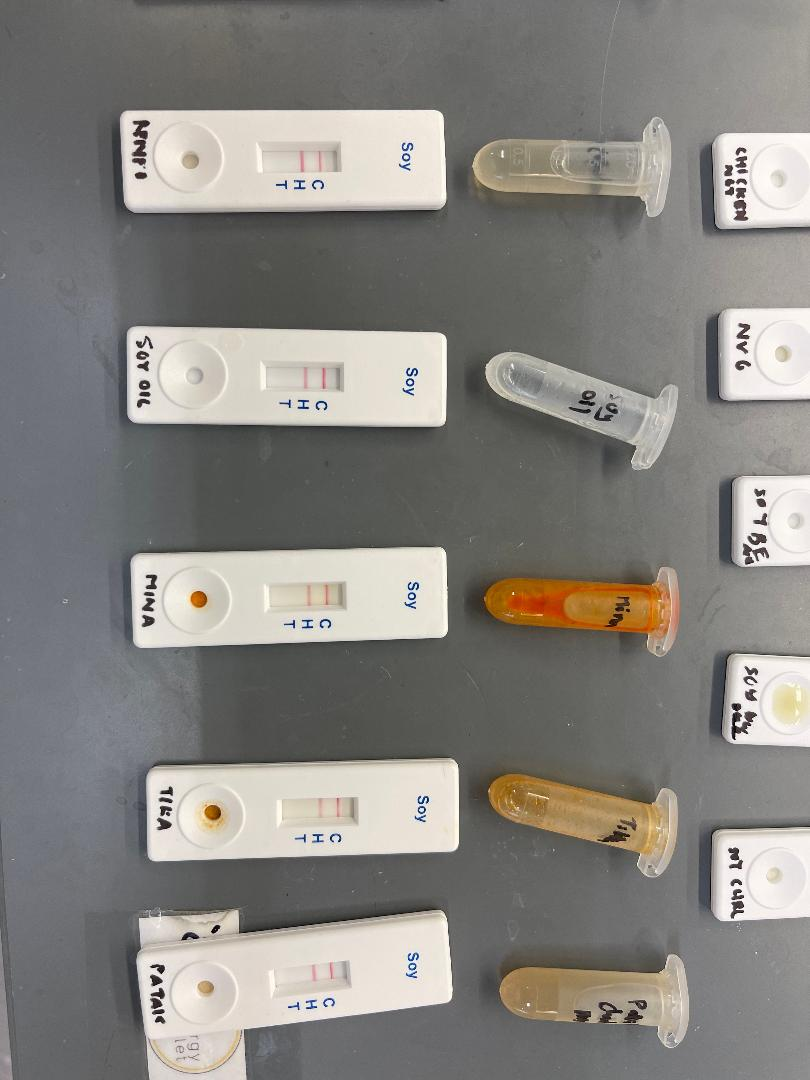


**B**

**A**

**C**

**D**

**E**

**F**

**G**

**H**

**I**

**J**

**K**

**L**


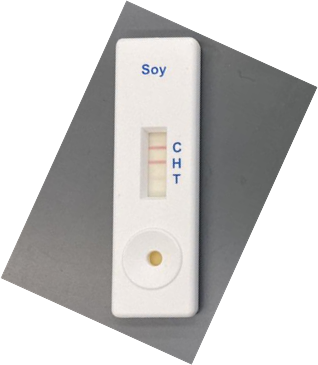


# **Figure S5**. LFDs data from soy protein isolate (**A**), Chicken (Not!) (**B**), soy curl (**C**), tofu (**D**), soybean (**E**), soy flour (**F**), 4X dilution soy flour (**G**), soy sauce (**H**), soybean oil (**I**), soy lecithin (**J**), 10X dilution soy lecithin (**K**), and Toast Chee peanut butter crackers (**L**).

# **Figure S6.** DPV responses recorded for soy allergen tracer (red line), juglone (blue line), chrysin (purple line), catechin (green line), and amygdalin (gold line) at 10 ppm concentration in 10% ethanol and 90% PBS 1X.


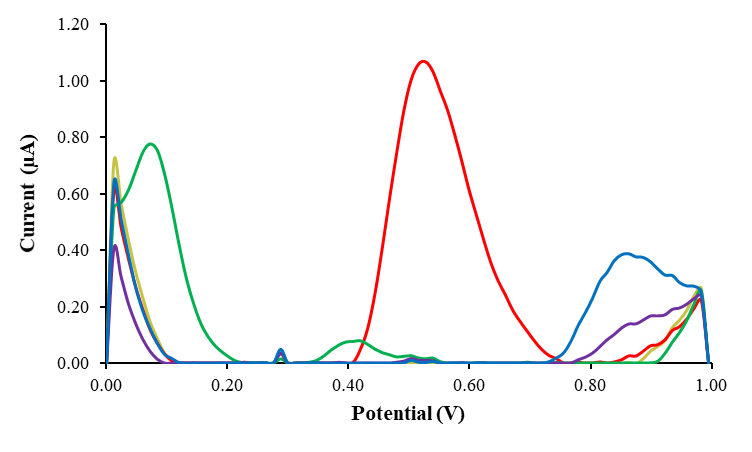


**Juglone**

**Chrysin**

**Amygdalin**

**Catechin**

**Soy allergen tracer**

To confirm the specificity of the sensor, we tested for cross-reactivity using interferent molecules that are structurally similar to the soy allergen tracer. We selected four compounds: amygdalin, juglone, chrysin, and catechin, which are commonly found in foods such as walnuts, honey, tea, and cocoa. The DPV measurements of the sensor in the juglone, chrysin, and catechin solutions showed oxidation peaks at 0.9 V, 0.9 V, and 0.1 V, respectively (**Figure S6**). Amygdalin did not show redox activity in the investigated potential range. On the contrary, the soy allergen tracer exhibited a well-defined redox peak at 0.6 V vs. Ag/AgCl reference electrode. The differences in redox activity between investigated species allowed us to distinguish soy allergen tracer from several structurally analogous interferents. In addition, we observed that the presence of interfering molecules had minimal impact on the soy allergen tracer peak current intensity when both species are present in the same sample solution. These findings indicate high selectivity of the fabricated MIP-based sensors for detecting the soy allergen tracer.

# **
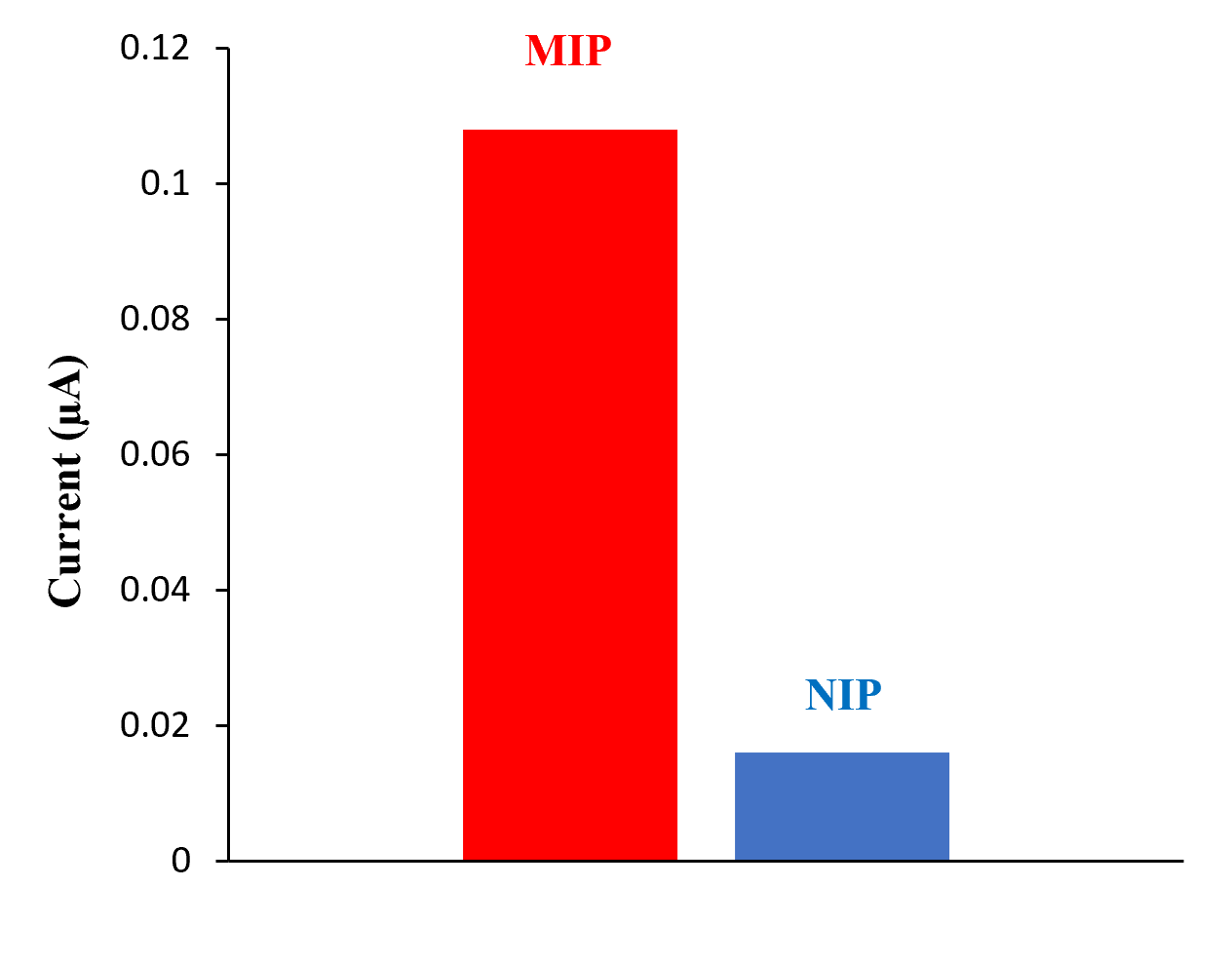
Figure S7.** DPV peak currents for the MIP and NIP in 10 ppm of the soy allergen tracer in 10% ethanol and 90% 1X PBS.

The selectivity of the MIPs was measured by the imprinting factor, the ratio of the DPV peak current for the MIP to the DPV peak current for the NIP. The imprinting factor for the 10 ppm genistein solution was 6.8, indicating that the soy allergen tracer was successfully incorporated into the film.
